# Variability of Glucosinolates in Pak Choy (*Brassica rapa* subsp. *chinensis*) Germplasm

**DOI:** 10.3390/plants13010009

**Published:** 2023-12-19

**Authors:** Seong-Hoon Kim, Kingsley Ochar, Aejin Hwang, Yoon-Jung Lee, Hae Ju Kang

**Affiliations:** 1 National Agrobiodiversity Center, National Institute of Agricultural Sciences, Rural Development Administration, Jeonju 5487, Republic of Korea; ocharocharking@yahoo.com (K.O.); hyj6138@korea.kr (A.H.); yoon112@korea.kr (Y.-J.L.); 2 Council for Scientific and Industrial Research, Plant Genetic Resources Research Institute, Bunso P.O. Box 7, Ghana; 3 Department of Agrofood Resources, National Institute of Agricultural Sciences, Rural Development Administration, Wanju 55365, Republic of Korea; kanghaeju5@korea.kr

**Keywords:** Brassica, compounds, germplasm, glucosinolates, pak choy, phytochemicals

## Abstract

Glucosinolates are sulfur-containing phytochemicals generally abundant in cruciferous vegetables such as pak choy. Glucosinolates participate in a range of biological activities essential for promoting a healthy human body. In this study, we aimed to elucidate glucosinolate variability present in pak choy germplasm that are under conservation at the Rural Development Administration Genebank, Jeonju, Republic of Korea. The Acquity Ultra-Performance Liquid Chromatography (UHPLC) analytical system was used in profiling the glucosinolate content in leaf samples of various accessions. We identified a total of 17 glucosinolates in the germplasm. Based on principal compoment analysis performed, three separate groups of the accessions were obtained. Group 1 contained the cultivar cheongsacholong which recorded high content of glucobrassicin (an indole), glucoerucin (aliphatic), gluconasturtiin (aromatic) and glucoberteroin (aliphatic). Group 2 consisted of six accessions, BRA77/72, Lu ling gaogengbai, 9041, Wuyueman, RP-75 and DH-10, predominatly high in aliphatic compounds including glucoiberin, glucocheirolin, and sinigrin. Group 3 comprised the majority of the accessions which were characterized by high content of glucoraphanin, epiprogoitrin, progoitrin, and glucotropaeolin. These results revealed the presence of variability among the pak choy germplasm based on their glucosinolate content, providing an excellent opprtunity for future breeding for improved glucosinolate content in the crop.

## 1. Introduction

Plant bioactive compounds or phytochemicals continue to attract extensive multidisciplinary research attention due to their known essential roles in the human body [1,2]. In particular their antioxidant prospects and consequently their involvements in many human disease prevention therapies make these compounds worthy of research [3,4]. A broad diversity of phytochemicals is reported in fruit and vegetable species which underscores the importance of consuming diets that are rich in essential bioactive compounds [5,6]. Pak choy (*Brassica rapa* subsp. *chinensis*), a member of the family Brassicaceae, is widely recognized as an important leafy vegetable not only for its remarkable culinary adaptability and nutritional richness but also its association with health benefits in humans [7,8]. Pak choy has characteristic non-heading leaves, which makes the crop distinct from its close relatives, mainly Chinese cabbage and yellow sarson [9,10]. As a result of the presence of a wide range of variability in the crop such as its morphological features, there are diverse accessions of pak choy found in many major Genebanks across the globe [9]. As a cruciferous vegetable, pak choy contains glucosinolates (GSL), a category of sulfur-containing secondary plant metabolites that have come under intense scrutiny for their bioactive properties and potential health-enhancing effects [11,12]. GSLs are famous based on their linkage with a broad range of health benefits such as their antioxidant, antimicrobial, and anti-inflammatory activities, and perhaps most notably their anticancer properties [12,13,14]. By examining GSLs within pak choy germplasm, researchers can gain a thorough understanding of their composition and concentrations across different genotypes and populations [15,16]. Such a comprehensive chemical profiling facilitates the identification of germplasm with high glucosinolate content. Germplasm containing GSLs at a desirable level of composition can be leveraged to develop functional foods or dietary strategies aimed at harnessing specific health benefits [17]. This further presents a treasurable resource for research aimed at investigating the composition and variations of GSLs present in different accessions of the crop for the purpose of crop improvement.

Technological advancements, particularly in the domain of analytical techniques like Ultra-Performance Liquid Chromatography-Tandem Mass Spectrometry (UPLC) integrated with mass spectrometry (MS), have considerably increased the precision and accuracy level of GSLs analyses [18,19]. Such cutting-edge techniques ease the identification, separation, and quantification process of the numerous GSLs present in pak choy tissues. The enhanced ability to accurately measure and quantify GSLs leads to comprehensive profiling, fostering a profound understanding of the GSL content in pak choy germplasm [20,21]. We are of the view that investigating GSL content in pak choy germplasm stored in Genebanks coincides with the growing global interest in enhancing nutritionally rich diets and promoting the consumption of functional foods [22,23]. By extracting the composition and assessing the presence of variability of GSLs in pak choy, researchers can help devise strategies that maximize the potential health benefits associated with these bioactive compounds. Therefore, in this study, we profiled GSLs content in pak choy germplasm selected from the Rural Development Administration genebank (RDA-Genebank), the Republic of Korea using the Acquity Ultra-Performance Liquid Chromatography (Milford, CT, USA) [24]. We analyzed the compositional variation in the compounds and identified accessions that exhibited desirably high GSLs contents, thus providing substantial insights for breeders. Overall, our results confirmed that the information on GSLs profiled in this study is useful for metabolic differentiation within the pak choy germplasm. Detailed knowledge about the diversity of GSLs in pak choy will shed light on the phytochemical composition of this vegetable; also, the study underscores the potential of the compound as a functional food with significant health implications, emphasizing the importance of incorporating this vegetable into a balanced diet for disease prevention and overall well-being.

## 2. Materials and Methods

### 2.1. Chemicals and Glucosinolate Standards

We used analytical-grade chemicals sourced from ThermoFisher Scientific Korea Ltd. (Seoul, Republic of Korea) and Sigma-Aldrich (St. Louis, MO, USA) for the extraction and analyses of the GSLs. Seventeen commercially available GSL standards used for the analyses were procured either from Phytoplan (Neuenheimer, Heidelberg, Germany) or Phytolab (Martin Baue, KG, Vestenbergsgreuth, Germany). The entire GSL standards had a purity ≥98%. Table 1 shows details of GSL standards used in this study.

### 2.2. Plant Materials, Sample Preparation and Compound Extraction

The National Agrobiodiversity Center of the Rural Development Administration (RDA), Jeonju, Republic of Korea has over 90 pak choy accessions under conservation in its National Genebank (RDA-Genebank). In this study, we selected 65 different pak choy accessions, classified as either cultivar or landrace, originating from different geographical locations (Table S1). First, seeds of the various accessions were multiplied under greenhouse conditions from February to June, 2019–2021. High-quality seeds from the various pak choy accessions were then selected for planting in the experimental field of the National Institute of Agricultural Sciences, Jeonju, and Republic of Korea form September to November 2021. For each accession, leaves of 18 plants, all of uniform phenotype, were collected, pooled together and kept in polyvinyl bags and stored at −80 °C until they were used for GSL extraction. The mixed leaf samples per each of three replications were analyzed. The extraction of the GSLs was performed based on the procedure as used previously in the work of Kim et al. [24]. Briefly, a 0.1 g sample containing 5 mL methanol (80%) that had been stored in a temperature of 25 °C for 30 min was thoroughly shaken to mix the contents at 120 rpm for 30 min at room temperature. The mixture was centrifuged (14,000 rpm at 4 °C for 10 min) and the supernatants transferred to fresh vials for GSL analyses using UPLC-MS/MS.

### 2.3. Identification and Quantification of GSLs Using UPLC-MS/MS

The Acquity Ultra-Performance Liquid Chromatography (UPLC) System (Waters, Milford, CT, USA) provides an advanced level of chromatographic performance for the separation and detection of compounds [25]. This system reduces the likelihood of undetected analytes, consequently ensuring increased efficiency and confidence of results [20,21]. In this experimental study, analyses of the GSL content of each sample was performed using the Acquity UPLC System coupled to a Xevo™ TQ-S system (Waters, MS Technologies, Wilmslow, UK). Here, 5 µL of the GLS sample was analyzed with the Acquity UPLC BEH C18 (1.7 µm, 2.1 × 100 mm) column. For the elution, 0.1% trifluoroacetic acid in water was used as Eluent A, with Eluent B mobile phase of 0.1% trifluoroacetic acid in methanol. The flow rate was maintained at 0.5 mL/min, column temperature at 35 °C and injection volume of 5 µL. The elution conditions were set as 100% of A from 0.0 to 1.0 min, 100% of A from 1.0 to 7.0 min, 100–80% of A from 7.0 to 10 min, 80–0% of A from 10 to 11 min, 0–100% of A from 11 to 15 min, and 100% of A thereafter. For the detection of the GSLs, negative ion electrospray ionization (ESI-) and multiple reaction monitoring (MRM) modes were employed. The MS/MS parameters were set using capillary and con voltages set at 3 kV and 54 V, respectively, for ionization. The ionization source was set at a 150 °C temperature while the dissolution temperature was set at 350, 150 °C. For cone and dissolution gas, temperature was set at 150 and 650 Lh-, respectively. The identification of the GSLs was carried out through direct comparison involving the retention times and MS and MS/MS fragmentation spectra with the commercially procured standards. We measured the linear, intraday, and interday precision in order to validate the precision and accuracy of the method used. For standards preparation, 10 mg of each GSL was dissolved in methanol to obtain stock solutions (1 mg mL^−1^). To calculate GSL concentrations, calibration curves were plotted based on the corresponding standards and the results expressed as µmol GSLs kg^−1^ sample dry weight (DW). The LOD (limit of detection) and LOQ (limit of quantification) values were taken as three and ten times, respectively, the standard error of the intercept of the regression equation of the linear calibration curve divided by the slope (Table 2). Fresh batches of test solutions were always prepared before sample analysis.

### 2.4. Statistical Analysis

All the analyses were performed using three independent samples as biological replicates. The resulting data were subjected to analysis of variance (ANOVA) using the XLSTAT software v2019 (Addinsoft, Paris, France). The quantification data obtained were used for principal component analysis. The Pearson’s correlation coefficient method was used to visualize the association of GSL compounds in the data. To obtain optimal clustering, we employed the K-means method to present a dendrogram. We also used the Orthogonal Partial Least Squares Discriminant Analysis (OPLS-DA), first to study the distribution and second to identify the key variables of GSL compounds responsible for cluster differentiation.

## 3. Results and Discussion

### 3.1. Variability of GSL Metabolite Composition in Pak Choy Germplasm

Glucosinolate compounds are famous for their potential health benefits. The breakdown of GSLs provides many useful products such as isothiocyanates, indoles, and sulforaphane which are associated with several biological activities in the human body [26,27]. The composition of GSLs in pak choy naturally differs across different geographical regions as well as on the basis of their type, such as cultivar or landrace. Details of the raw data of GSL content of the accessions used in the current study would be made available and accessed from the RDA-Genebank at http://genebank.rda.go.kr/. In order to satisfy our curiosity on the presence of variability of GSL content in pak choy, we employed the Acquity Ultra-Performance Liquid Chromatography-Tandem Mass Spectrometry (UPLC-MS/MS) analysis system to determine the GSL composition of diverse pak choy accessions that are under conservation in the national gene bank of the Rural Development Administration (RDA-Genebank), Jeonju, Republic of Korea. Our analyses detected a total of 17 glucosinolate-derived metabolites in the germplasm. Details of the Acquity UPLC spectroscopy analysis results of the 17 GSLs are shown in Table 3. In a previous study, Ju-Hee and co-workers [28] identified eight GSLs based on quantification of leaf samples from five commercial varieties and 45 accessions of diverse Brassica species including Kimchi cabbage, turnip and leaf mustard. Earlier, using 13 pak choy cultivars, Wiesner and colleagues [26] detected 11 GSLs that were predominantly composed of members of the aliphatic class. We examined the composition of the GSLs in the 65 pak choy accessions by calculating their concentration expressed in median values (µmol·kg^−1^ DW). Generally, significant variability was observed across the different classes of the GSLs (aliphatic, aromatic and indolic) (Table 3). The most predominant GSLs detected were in the aliphatic class. In line with a previous study by Wiesner et al. [26] in which pronounced variation in total aliphatic GSLs (18.7–61.7 μmol g^−1^ dw) was observed, we found significant variation among the aliphatic GSLs in the present study. A similar finding was presented in another study in which aliphatic compounds represented the predominant GSLs detected [28].

In this study, among the aliphatic GSLs, Gluconapin (GNA) recorded the highest concentration (median: 6713.083 µmol·kg^−1^ DW), while Glucoraphasatin represented the lowest concentration (median: 0.231 µmol·kg^−1^ DW). For the aromatic GSLs, Gluconasturtiin and Sinalbin recorded the highest and least concentrations with median values of 678.72 µmol·kg^−1^ DW and 0.086 µmol·kg^−1^ DW, respectively. An indolic GSL, Glucobrassicin had a concentration of 351.011 µmol·kg^−1^ DW higher than 50% and 75% of the aromatic and aliphatic compound, respectively, contrasting with that reported by Wiesner et al. [26], where indolic GSL occurred at low levels (0.6 to 2.35 µmol·g^−1^ dw). This contradiction perhaps resulted from differences in pak choy genetic materials as well as the extraction protocol used [28,29].

Previous studies on GSLs from various *B. rapa* vegetables revealed that choi sum has high GNA content compared to other *Brassica* vegetables [12,30]. However, the least detected GSL was GBC according to He and co-workers [30], contrasting with the Sinalbin (SNB) compound that is observed in our current study [31]. Clear variations in GSL content and individual GSL profiles exists among *Brassica* vegetables [30,32]. For example, leaf mustard (*B. juncea*), turnips (*B. rapa* var. rapa), collards (*B. oleracea* var. *viridis*), and kale (*B. oleracea* var. *sabellica*) exhibit higher total GSL levels compared to pak choy and choy sum (*B. chinensis* var. *parachinensis*) [30]. While aliphatic GSLs are generally abundant in *Brassica* species [33], choi sum and Chinese cabbage mainly contain GNA and GBN [24]. Pak choy, on the other hand, is very rich in GNA and PRO among aliphatic GSLs (Table 3). Furthermore, Gluconasturtiin displayed the highest median value (678.72 µmol·kg^−1^ DW) among aromatic GSLs. Glucotropaeolin and Glucobarbarin also demonstrated significant median values of 6.451 and 3.171 µmol·kg^−1^ DW, respectively. Glucobrassicin, the only compound detected as an indolic GSL in this study exhibited appreciably high median value (351.011 µmol·kg^−1^ DW).

Our results highlight promising genetic resources, offering breeders advanced cultivars to develop nutritionally superior vegetables in pak choy breeding programs. In the present finding, IT23558 had the highest GNA content with a median value of 19,009.9 µmol·kg^−1^ DW. This value surpassed the average GNA content in choy sum (*B. chinensis* var. *parachinensis*) by a notable margin of 2997.62 µmol·kg^−1^. This germplasm, collected in 2001 from the RDA-Genebank to enhance genetic diversity, is available for purchase in the Republic of Korea. However, due to absence of detailed passport information, the origin or country of the breeding company remains unclear. Along with its high GNA content, this cultivar also contains progoitrin (PRO) levels of 2483 µmol·kg^−1^ DW, approximately twice the median value. PRO is recognized for its anti-inflammatory properties. As demonstrated by Jang and colleagues [31], it has increased antibacterial activity against Aeromonas hydrophilic [18]. Yet, it is crucial to note that PRO levels exceeding 3000 µmol·kg^−1^ DW can introduce a bitter taste, potentially affecting palatability and hindering growth in certain animals [34]. This might require selective use, depending on breeding objectives. The germplasm with the second highest GSL content, IT275755 (9039), contained 18,268.9 µmol·kg^−1^ DW. Cultivated in 2013 by the Asia Seed Company (Seoul, Korea), this germplasm was subsequently deposited at the RDA-Genebank. Morphologically, it is characterized as an overwintering type and developed as a red-headed cabbage cultivar. Its foliage is green, with a mid-season bolting pattern, displaying a dwarf growth habit. Significantly, its PRO content is exceptionally low at 5.993 µmol·kg^−1^, signifying reduced bitterness, a distinguishing trait of this cultivar.

### 3.2. Multivariate Analysis

#### 3.2.1. Correlation Analysis

**Figure 1:**
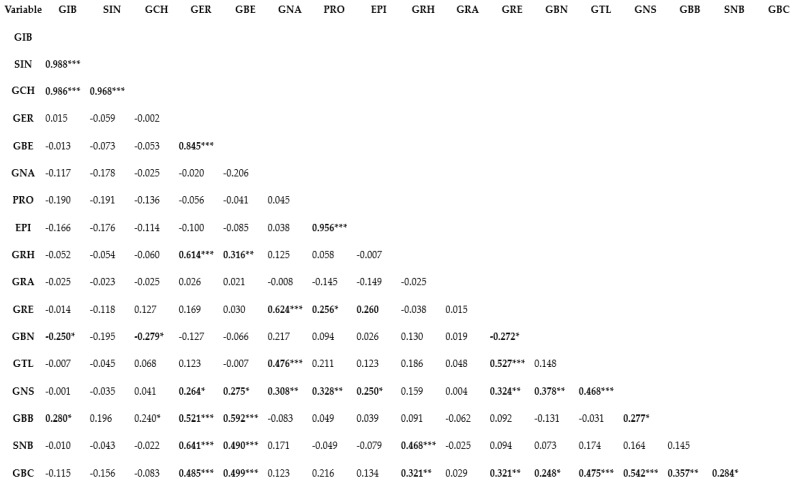
Pearson’s correlation analysis of glucosinolates detected in pak choy. ***, **, * indicate correlation is significant at *p* ≤ 0.0001, *p* ≤ 0.01, and *p* ≤ 0.05, respectively. Glucoiberin (GIB), Sinigrin (SIN), Glucocheirolin (GCH), Glucoerucin (GER), Glucoberteroin (GBE), Gluconapin (GNA), Progoitrin (PRO), Epiprogoitrin (EPI), Glucoraphasatin (GRH), Glucoraphanin (GRA), Glucoraphenin (GRE), Glucobrassicanapin (GBN), Glucotropaeolin (GTL), Gluconasturtiin (GNS), Glucobarbarin (GBB), Sinalbin (SNB)and Glucobrassicin (GBC).

Genetic correlation represents the relationship between two variables in a data set and provides a clue to the understanding of the shared biological network or the magnitude of association between variables [4,35]. Understanding the correlation or association between traits is important in deciding whether or not selection for a specific trait will influence the other [36,37]. Correlation analysis can be applied conveniently in determining the relationships among different phytochemical compounds [38,39]. Pearson’s correlation coefficient (r) is used in measuring the linear association of two variables and has been used for establishing the association between different bioactive compounds [4,40]. In this study, the Pearson’s correlation coefficient (r) of GSLs was estimated using the XLSTAT analysis software v.2019 (Addinsoft, Paris, France) (Figure 1). A very strong positive association characterized a number of aliphatic compounds including SIN and GIB (r = 0.988; *p* ≤ 0.0001), GCH and GIB (r = 0.986; *p* ≤ 0.0001), GCH and SIN (r = 0.968; *p* ≤ 0.0001), GBE and GER (r = 0.845; *p* ≤ 0.0001) and EPI and PRO (r = 0.956; *p* ≤ 0.0001). A positive association between two GSL indicates that the two variables or compounds are located in biologically tightly linked pathways, and as a result of their shred biochemical nature, these compounds likely share the same cluster [4]. Thus, in crop breeding, indirect selection or improvement of one GSL compound could simultaneously contribute to enhancing the content of the other [41]. Similarly, a positive correlation was observed between GRH and GER (r = 0.614; *p* ≤ 0.0001), SNB and GER (r = 0.641; *p* ≤ 0.0001), and GRE and GNA (r = 0.624; *p* ≤ 0.0001). Other noticeable positive associations were observed between GBS and GER (R = 0.485; *p* ≤ 0.0001), SNB and GBE (r = 0.490; *p* ≤ 0.0001), SNB and GRH (r = 0.468; *p* ≤ 0.0001), GBC and GBE (r = 0.499; *p* ≤ 0.0001), GTL and GNA (r = 0.476; *p* ≤ 0.0001), GTL and GRE (r = 0.527; *p* ≤ 0.0001), GNS and GTL (r = 0.468; *p* ≤ 0.0001), GBC and GTL (r = 0.475; *p* ≤ 0.0001), GCB and GNS (r = 0.542; *p* ≤ 0.0001). The above results reveal that Brassica genotypes containing higher amount of one or more of the GSLs could be used in crop breeding [42] to increase the composition of other compounds since the compounds are likely to be biosynthetically linked. In correlation analyses, two variables display a negative correlation coefficient suggesting that indirect selection or improvement of one of the variables does not potentially contribute to enhanced expression of the other. In this study, generally, GSL compounds that are negatively correlated had weak correlation coefficient (Figure 1). The highest negative correlation coefficient was observed between GBN and GIB (r = −0.250; *p* ≤ 0.05), GBN and GCH (r = −0.279; *p* ≤ 0.05). Thus, genetic improvement of one of GBNs with the aim to enhance the composition of GIB or GCH is likely less useful.

#### 3.2.2. Variability of GSLs in Pak Choy Based on PCA

**Figure 2:**
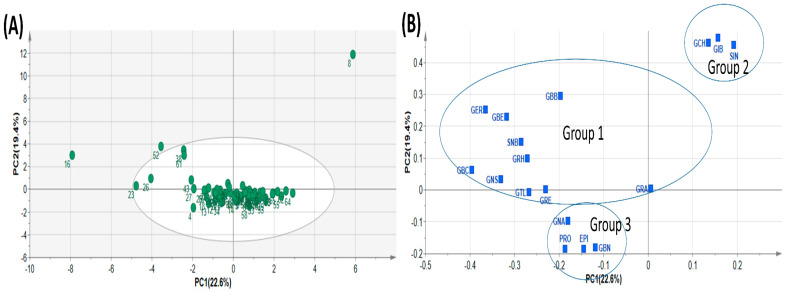
Principal Component Analysis showing Score plot of the 65 pak choy accessions (**A**) and Loading plot of the 17 pak choy accessions (**B**). Glucoiberin (GIB), Sinigrin (SIN), Glucocheirolin (GCH), Glucoerucin (GER), Glucoberteroin (GBE), Gluconapin (GNA), Progoitrin (PRO), Epiprogoitrin (EPI), Glucoraphasatin (GRH), Glucoraphanin (GRA), Glucoraphenin (GRE), Glucobrassicanapin (GBN), Glucotropaeolin (GTL), Gluconasturtiin (GNS), Glucobarbarin (GBB), Sinalbin (SNB) and Glucobrassicin (GBC).

**Figure 3:**
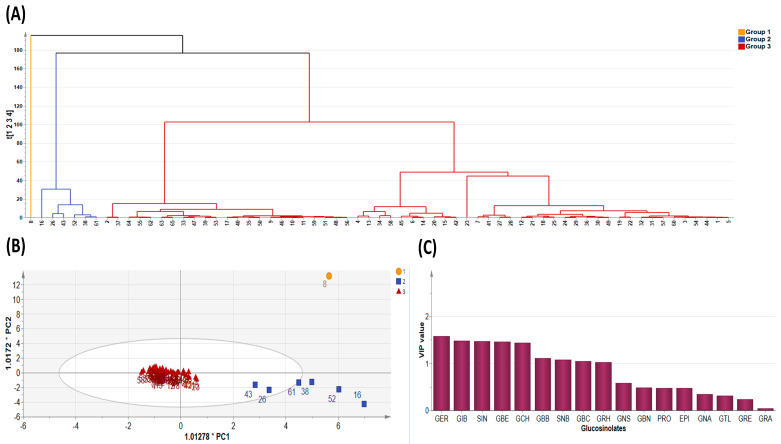
Diversity Analysis showing (**A**) cluster dendrogram of the 65 pak choy accessions, (**B**) Orthogonal Partial Least Squares Discriminant Analysis, and (**C**) Variable Importance in Projection value. Glucoerucin (GER), Glucoiberin (GIB), Sinigrin (SIN), Glucoberteroin (GBE), Glucocheirolin (GCH), Glucobarbarin (GBB), Sinalbin (SNB), Glucobrassicin (GBC), Glucoraphasatin (GRH), Gluconasturtiin (GNS), Glucobrassicanapin (GBN), Progoitrin (PRO), Epiprogoitrin (EPI), Gluconapin (GNA), Glucotropaeolin (GTL), Glucoraphenin (GRE) and Glucoraphanin (GRA).

Principal component analysis (PCA) is a typical chemometric tool commonly used in multivariate analysis for extracting and interpreting experimental results [4,43]. In order to investigate diversity in the GSL composition within the analytes, the quantification data of the 17 GSL compounds detected in the study were subjected to principal component analysis. PCA was used to determine the most relevant components with the largest variation. The data dimension was reduced to three principal components by employing the eigenvector values greater than or equal to one. The first three principal components accounted for 56.567% of the cumulative total variation (Table 4). PC1 accounted for 22.604% of the total variation while PC2 and PC3 accounted for 19.373 and 14.591% of the total variance, respectively. The two highest ranking components PC1 and PC2) accounted for 41.976% of the total variance with eigenvector values 3.843 and 3.293, respectively, lower than those obtained in some previous studies. For instance, Wiesner et al. [26] recorded 86% of total variation derived from the first three PCs, with the highest ranking PCs, PC1 and PC2, accounting for 49% and 22%, respectively. Generally, the PCs had many positive loading relative to the negative loadings (Table 4). The highest positive loading in PC1 corresponded with GBC (0.395), GER (0.365), GNS (0.331), and GBE (0.317), representing all the three classes of compounds. PC2 showed strong positive loading with three aliphatic GSL compounds, GIB (0.478), GCH (0.461) and SIN (0.455), which mainly accounted for the variability in GSLs profiled in this experiment. PCA revealed three distinct groups of all the pak choy accessions (Figure 2A and Figure 3A,B) and the GSL compounds (Figure 2B), suggesting the presence of variability in GSLs among the genetic materials used in the study. A similar finding was previously reported in the works of Wiesner and colleagues who found aliphatic glucosinolates as the predominant GSL class in pak choi [26]. Nonetheless, the unexplained 44% of variability of the glucosinolates may be due to additional underlying biochemical pathways or metabolites not captured by the three principal components. This unaccounted variation could also be a result of the influence of environmental factors or complex interactions among multiple metabolites not emphasized by the primary components. Therefore, further investigations, potentially through targeted analyses or additional statistical methods, are necessary to elucidate the specific contributors to this remaining variance.

Since PCA is limited by accuracy of clustering, we employed the K-means method to obtain optimal clustering [44] and this was presented as a dendrogram of three clusters (Figure 3A). By its dimension reduction property, the Orthogonal Partial Least Squares Discriminant Analysis (OPLS-DA) is more suitable for sample distinction relative to PCA [45]. So, in order to gain additional insight into the diversity of pak choy germplasm, the OPLS-DA was used to identify the key accessions that contributed to the cluster differentiation based on Variable Importance in Projection (VIP) values [46,47]. Here, three separate groups of pak choy were obtained as indicated with a yellow circle, a blue rectangle and a red triangle (Figure 3A,B). Group 1 contained the Korean cultivar cheongsacholong which recorded high content of GBC (indolic), GER (aliphatic), GNS (aromatic) and GBE (aliphatic) (Figure 2B and Figure 3A,B). Conversely, this accession recorded lower levels of GIB, SIN, GCH, and GRA, all of which are aliphatic compounds. Group 2 consisted of six accessions, BRA77/72, Lu ling gaogengbai, 9041, Wuyueman, RP-75, DH-10, which were predominatly high in aliphatic compounds including GIB, GCH, and SIN. Also, these accessions recorded lower levels of aliphatic compounds GNA, PRO, EPI, GBN, and GTL. Group 3 comprised the majority of the accessions (Figure 2A and Figure 3A,B). This group of accesstions showed a characteristic high content of aliphatic GSLs encompassing GRE, EPI, PRO, and GTL, but low content of GER, GBE, GRH, GRA, GBN, GBB and SNB. These results reveal the presence of distinct variation among the pak choy germplasm based on their GSL content, providing an excellent opprtunity for future breeding for improved GSL content in pak choy using reommended accesions [48]. The presence of diversity among pak choy accessions was reported in other studies [26,49]. Figure 3C indicates individual GSLs and their contibutions to the three clusters based on VIP values. According to Park et al. [50], in a given data set, variables with VIP values greater than one are the most significant contributors to the observed variablity. Based on the VIP values, the most influencial class of compounds responsible for variation in the germplasm were mainly attributed to nine metabolites including six aliphatic (GER, GIB, SIN, GBE, GCH and GRH), two aromatic (GBB and SNB) and one indolic (GBC) compounds (Figure 3C).

Overall, our results confirmed that the GSL compounds profiled in this study provide useful information for metabolic differentiation within the pak choy germplasm and provided useful information to facilitate breeding in pak choy.

## 4. Conclusions

In this study, we identify 17 GSLs among 65 pak choy accessions, indicating the presence of variability of the compound within pak choy germplasm. The identification and characterization of various GSLs, along with their potential health benefits, highlight the importance of pak choy as a valuable functional food with significant implications for human health. In the future, there is the need for further research on GSLs profiles in pak choy in relation to other cruciferous vegetables. Additionally, further investigations are required in elucidating the precise mechanisms through which these GSLs exert their health-promoting effects, particularly their roles in cancer prevention, anti-inflammatory activity, and detoxification pathways within the body. Studies are also needed to bridge the gap between laboratory analyses and practical applications, seeking methods to optimize GSL content in pak choy through agricultural practices or breeding strategies. This can enhance the nutritional value of the crop, thus increasing the crop’s potential health benefits for consumers. Still, additional studies are warranted to understand the impact of cultivation conditions, and processing methods on the levels and types of these bioactive compounds in pak choy. Overall, the exploration of GSLs diversity in pak choy germplasm provides a promising avenue for harnessing the health benefits of these phytochemicals. The present study provides a basis for future research directions aimed at maximizing the potential of pak choy as a functional food for promoting human health and well-being.

## Figures and Tables

**Table 1:** List of glucosinolate standards procured and used in the study.

Class	Glucosinolate	Abbreviation	Molecular Formula	Molecular Weight (g/mol)	Source
Aliphatic GSL	Glucoiberin	GIB	C11H21NO10S3	423.5	Phytolab
	Sinigrin	SIN	C10H16KNO9S2	397.5	Phytoplan
	Glucocheirolin	GCH	C11H20KNO11S3	477.6	Phytoplan
	Glucoerucin	GER	C12H23NO9S3	421.5	Phytoplan
	Glucoraphanin	GRA	C12H23NO10S3	437.5	Phytoplan
	Gluconapin	GNA	C11H10NO9S2	373.4	Phytoplan
	Progoitrin	PRO	C11H19NO10S2	389.4	Phytolab
	Epiprogoitrin	EPI	C11H19NO10S2	389.4	Phytolab
	Glucoraphasatin	GRH	C12H21NO10S3	435.5	Phytoplan
	Glucoraphanin	GRE	C12H23NO10S3	437.5	Phytolab
	Glucoberteroin	GBE	C13H25NO9S3	435.5	Phytoplan
	Glucobrassicanapin	GBN	C12H21NO9S2	387.4	Phytolab
Aromatic GSL	Glucotropaeolin	GTL	C14H19NO9S2	409.4	Phytoplan
	Gluconasturtiin	GNS	C15H21NO9S2	423.5	Phytoplan
	Glucobarbarin	GBB	C15H21NO10S2	439.5	Phytoplan
	Sinalbin	SNB	C14H19NO10S2	425.4	Phytolab
Indolic GSL	Glucobrassicin	GBC	C16H20N2O9S2	448.5	Phytoplan

**Table 2:** Results of the UPLC spectroscopy analysis showing the seventeen glucosinolates, retention time (RT), calibration curves, and multiple reaction monitoring (MRM) conditions for quantitation of glucosinolates by negative ion MRM.

Class	Name	Abbreviation	RT (min)	MRM Transition	CID (ev)	Dwell Time (sec)	Calibration Curve Parameters
Aliphatic	Progoitrin	PRO	5.94	387.77 > 194.85	25	0.029	Y = 8.2526X + 28.1501 (*r*^2^ = 0.961)
	Sinigrin	SIN	6.56	357.75 > 161.84	25	0.029	Y = 12.7878X − 11.1181 (*r*^2^ = 0.999)
	Gluconapin	GNA	7.78	371.74 > 258.74	25	0.029	Y = 8.36216X + 29.5397 (*r*^2^ = 0.994)
	Glucoiberin	GIB	7.98	421.62 > 357.73	25	0.029	Y = 33.6632X + 446.334 (*r*^2^ = 0.997)
	Epiprogoitrin	EPI	8.06	387.7 > 258.74	25	0.029	Y = 7.4939X − 6.76519 (*r*^2^ = 0.999)
	Glucocheirolin	GCH	8.38	437.71 > 258.74	25	0.029	Y =20.7762X + 39.3608 (*r*^2^ = 0.986)
	Glucoraphanin	GRA	8.39	435.59 > 177.78	25	0.029	Y = 25.0808X +60.584 (*r*^2^ = 0.983)
	Glucoraphenin	GRE	8.53	433.66 > 258.81	25	0.029	Y = 15.2565X + 3.62242 (*r*^2^ = 0.988)
	Glucobrassicanapin	GBN	8.60	385.71 > 258.87	25	0.029	Y = 7.2514X + 47.2841 (*r*^2^ = 0.992)
	Glucoerucin	GER	8.73	419.69 > 258.74	25	0.029	Y = 6.77393X + 73.6679 (*r*^2^ = 0.984)
	Glucoberteroin	GBE	9.18	433.72 > 275.06	25	0.029	Y = 6.09397X + 63.1212 (*r*^2^ = 0.997)
	Glucoraphasatin	GRH	9.62	417.63 > 258.81	25	0.029	Y = 15.5149X − 5.95281 (*r*^2^ = 0.997)
Aromatic	Glucobarbarin	GBB	8.64	437.71 > 274.75	25	0.029	Y = 9.29915X− 0.454779 (*r*^2^ = 0.999)
	Glucotropaeolin	GTL	8.88	407.72 > 258.87	25	0.029	Y = 18.2122X − 3.93949 (*r*^2^ = 0.999)
	Sinalbin	SNB	9.10	423.62 > 258.74	25	0.029	Y = 49.7228X − 33.0636 (*r*^2^ = 0.999)
	Gluconasturtiin	GNS	9.34	421.69 > 274.87	25	0.029	Y = 4.36109X − 90.233 (*r*^2^ = 0.961)
Indolyl	Glucobrassicin	GBC	9.31	446.69 > 204.94	25	0.029	Y = 6.39827X + 2.6232 (*r^2^* = 0.997)

**Table 3:** Profile of individual glucosinolates in pak choy (μmol∙kg^−1^ DW).

Class	Glucosinolates	Range	Median
Aliphatic GSL	Glucoiberin	0~35.069	0.375
	Sinigrin	0.162~7878.972	4.722
	Glucocheirolin	0.078~239.664	5.256
	Glucoerucin	0~2564.479	49.366
	Glucoraphanin	0.162~1558.413	172.591
	Gluconapin	117.379~19,009.896	6713.083
	Progoitrin	2.303~4116.955	1132.364
	Epiprogoitrin	1.629~3333.335	843.059
	Glucoraphasatin	0.025~6.134	0.231
	Glucoraphenin	0.0168~228.202	0.981
	Glucoberteroin	0~3491.342	148.188
	Glucobrassicanapin	0.263~8744.337	3139.729
Aromatic GSL	Glucotropaeolin	0.311~30.651	6.451
	Gluconasturtiin	74.282~2148.237	678.72
	Glucobarbarin	0.937~10.505	3.171
	Sinalbin	0~3.704	0.086
Indolic GSL	Glucobrassicin	77.984~1294.483	351.011

**Table 4:** Principal component analysis of GSL content in pak choy.

	Principal Component (Eigenvectors)		
GSL	PC1	PC2	PC3
GIB	−0.156	**0.478**	0.225
SIN	−0.192	**0.455**	0.210
GCH	−0.135	**0.461**	0.292
GER	**0.365**	0.251	−0.268
GBE	**0.317**	0.228	**−0.304**
GNA	0.180	−0.099	0.290
PRO	0.187	−0.187	**0.324**
EPI	0.145	−0.187	**0.327**
GRH	0.271	0.098	−0.167
GRA	−0.006	0.003	−0.064
GRE	0.230	0.001	**0.366**
GBN	0.119	−0.181	−0.007
GTL	0.268	−0.008	**0.320**
GNS	**0.331**	0.034	0.229
GBB	0.197	0.296	−0.053
SNB	0.285	0.150	−0.188
GBC	**0.395**	0.063	0.033
Eigen value	3.843	3.293	2.480
Proportion (%)	22.604	19.373	14.591
Cumulative (%)	22.604	41.976	56.567

The bold values represent the highest loadings in principal component analysis, which essentially accounted for the presence of variability in GSL profiles.

## Data Availability

The data used to support the findings of this study can be made available by the corresponding author upon request.

## Supplementary Materials

The following supporting information can be downloaded at: https://www.mdpi.com/article/10.3390/plants13010009/s1, Table S1: List of pak choy accessions used for GSL profiling.

## Abbreviations

GSL: glucosinolate; GIB, glucoiberin; SIN, sinigrin; GCH, glucocheirolin; GER, glucoerucin; GRA, glucoraphanin; GNA, gluconapin; PRO, progoitrin; EPI, epiprogoitrin; GRH, glucoraphasatin; GRE, glucoraphenin; GBE, glucoberteroin; GBN, glucobrassicanapin; GTL, glucotropaeolin; GNS, gluconasturtiin; GBB, glucobarbarin; SNB, sinalbin; GBC, glucobrassicin.
