# Supplementary material for: Variability of Glucosinolates in Pak Choy (Brassica rapa subsp. chinensis) Germplasm
Source: Plants (Basel). 2023 Dec 19;13(1):9. doi: 10.3390/plants13010009 (PMC10780573; doi:10.3390/plants13010009)
Supplement: Supplementary file 1 [file plants-13-00009-s001.zip › plants-2759633-supplementary.pdf]

Supplementary Data

Table S1. List of pak choy accessions used for GSL profiling

S/N	Accession	**IT No.**	Classification	Origin
1	AVRDC-KJH-1985-100364	100364	Landrace	TWN
2	AVRDC-KJH-1985-100375	100375	Landrace	TWN
3	AVRDC-KJH-1985-100403	100403	Landrace	TWN
4	Pai Tsai San-Yueh-Man	135443	Cultivar	CHN
5	Xiadongqing	191049	Cultivar	CHN
6	SawiPutih	221723	Landrace	MYS
7	Chinese Cabbage 15	221761	Landrace	CHN
8	cheongsacholong	223316	Cultivar	KOR
9	Jeogseolchae	223318	Cultivar	KOR
10	PT-21	227096	Cultivar	KOR
11	PT-22	227097	Cultivar	KOR
12	RP-21	227098	Cultivar	KOR
13	Chinese White Cabbage	227887	Landrace	CHN
14	Tientsin	227889	Landrace	CHN
15	Cabbage Tientsin, Large	227890	Landrace	CHN
16	BRA77/72	227891	Cultivar	CHN
17	Dwarf	227892	Landrace	CHN
18	CGN20729	227893	Landrace	TWN
19	CGN15187	227899	Landrace	TWN
20	Pakchoi white	235477	Landrace	UNK
21	MYS-CEG-1999-45	235480	Landrace	MYS
22	najjeusindokku	235556	Cultivar	JPN
23	josaenghwagyeong	235558	Cultivar	KOR

24	ching-gensai	235559	Cultivar	CHN
25	Zhang gengbaicai	247398	Cultivar	CHN
26	Lu ling gaogengbai	247399	Cultivar	CHN
27	8035	247776	Cultivar	KOR
28	8033	247778	Cultivar	KOR
29	THA-LSY-2000-141	247869	Landrace	THA
30	CHN-MKH-2001-99	247889	Landrace	CHN
31	Garak No. 1	259306	Cultivar	CHN
32	AS2	259414	Cultivar	NPL
33	MYS-PYJ-2007-97	260621	Landrace	MYS
34	PAK CHOY	262109	Landrace	MYS
35	Qing bang youcai	262111	Cultivar	CHN
36	9037	275754	Cultivar	KOR
37	9039	275755	Cultivar	KOR
38	9041	275756	Cultivar	KOR
39	9034	275757	Cultivar	KOR
40	Dongbeiqing	278547	Cultivar	CHN
41	MNG-2012-17	280035	Landrace	MNG
42	Quannianchunlvaijiaoba	280395	Landrace	CHN
43	Wuyueman	280396	Landrace	CHN
44	Siyueman	280397	Landrace	CHN
45	Eryueman	280398	Landrace	CHN
46	Neuheit	293031	Landrace	DEU
47	MYS-CGT-1999-69	293054	Landrace	MYS
48	MYS-CGT-1999-70	293055	Landrace	MYS
49	SHANHAIDA-BAITCA	293143	Landrace	CHN
50	SYYUSMAN	293144	Landrace	CHN

51	SHANHAI LEAGY	293234	Landrace	CHN
52	RP-75	297498	Cultivar	KOR
53	Green Flowering pak choy	299456	Cultivar	THA
54	Ai jiaodatouqing	301100	Cultivar	CHN
55	Low	301953	Landrace	CHN
56	Da touqing Jiang bai	301979	Landrace	CHN
57	Liuyueman	301980	Landrace	CHN
58	Sakushina	306672	Landrace	JPN
59	Chinchian	306720	Landrace	TWN
60	DH-5ms	308457	Cultivar	KOR
61	DH-10	308459	Cultivar	KOR
62	MYS-PYJ-2007-16	228226	Landrace	MYS
63	Shanidun No 4	-	Landrace	CHN
64	JP 26743	339595	Landrace	KOR
65	Qing gengxiaobaicai	339599	Cultivar	CHN
